# Impact of new-onset atrial fibrillation in patients with ST-segment elevation myocardial infarction

**DOI:** 10.1007/s10840-024-01941-5

**Published:** 2024-12-11

**Authors:** Judith Minder, Diego Mannhart, Sarah Brunner, Gianluca Di Bari, Sven Knecht, Philipp Krisai, Thomas Nestelberger, Jasper Boeddinghaus, Gregor Leibundgut, Christoph Kaiser, Christian Mueller, Stefan Osswald, Christian Sticherling, Michael Kühne, Patrick Badertscher

**Affiliations:** 1 Department of Cardiology, University Hospital Basel, Petersgraben 4, 4031 Basel, Switzerland; 2 Cardiovascular Research Institute Basel, University Hospital Basel, Petersgraben 4, 4031 Basel, Switzerland

**Keywords:** ST-segment elevation myocardial infarction, Atrial fibrillation

## Abstract

**Background:**

New-onset atrial fibrillation (NOAF) complicating ST-segment elevation myocardial infarction (STEMI) remains clinically challenging. The aim of this study was to assess the incidence of NOAF, identify risk factors for the development of atrial fibrillation (AF), and analyze the impact on patient care, therapy, and outcomes during long-term follow-up.

**Methods:**

This retrospective single-center study reviewed consecutive patients undergoing coronary angiography (CAG) for acute STEMI between May 2015 and September 2023. Patients were stratified in NOAF, defined as AF diagnosed during the index hospitalization or within 12 months of follow-up, AF prior to the hospitalization for STEMI, and patients with no AF.

**Results:**

We analyzed 1301 consecutive patients undergoing CAG for STEMI. NOAF was detected in 112 patients (9.8%), and 68 patients (5.2%) had prior AF. NOAF patients were 74% males, with a mean age of 69 ± 11 years. During a median follow-up of 683 days, the rates of stroke were 10% in patients with NOAF compared to 3.8% (*p* = 0.001) in patients without AF. Major bleeding occurred in 7% vs. 1.7%, *p* = 0.001, and death in 16% vs. 6.8%, *p* < 0.001 of patients with NOAF vs. no AF.

**Conclusion:**

NOAF was detected in almost 1 out of 10 STEMI patients and was associated with a higher rate of stroke, major bleeding, and death as in patients with no AF and with similar rates compared with prior AF. Future studies assessing optimal anticoagulation therapy in this challenging patient population are warranted.

**Graphical abstract:**

Central illustration: New-onset atrial fibrillation in patients with ST-segment elevation myocardial infarction: Inclusion criteria, stratification, and outcome data of AF and no AF patients in ST-segment elevation myocardial infarction. AF atrial fibrillation, NOAF new-onset atrial fibrillation

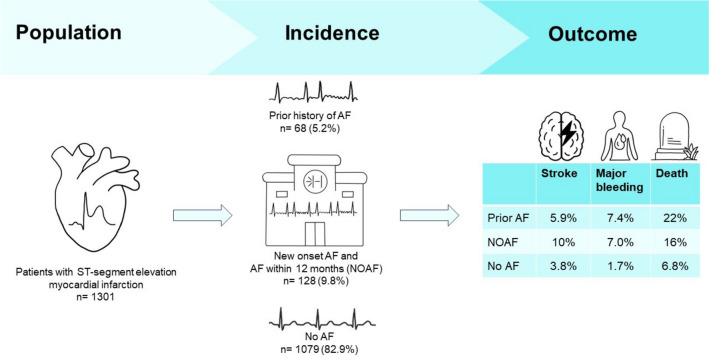

**Supplementary Information:**

The online version contains supplementary material available at 10.1007/s10840-024-01941-5.

## Introduction

Despite improvements in both pharmacological treatment and revascularization techniques, mortality among ST-segment elevation myocardial infarction (STEMI) patients remains high [1, 2]. The prognostic impact of the occurrence of cardiac arrhythmias during acute myocardial infarction (AMI) is unclear [3]. Among periprocedural cardiac arrhythmias, atrial fibrillation (AF) seems to be the most frequent [4].

While previous studies assessed the impact of known AF in STEMI patients [5–7], several questions remain for patients with new-onset atrial fibrillation (NOAF) in STEMI.

There is conflicting data regarding the incidence of NOAF in STEMI with incidences ranging from 2.3 to 36% [8–10]. Similarly to AF after non-cardiac surgery or during critical illness, patients seem to be vulnerable to AF during the immediate postprocedural period possibly due to transient factors such as inflammation, atrial ischemia, oxidative stress, or sympathetic activation [11–14]. These triggers indicate that NOAF after STEMI might be a transient phenomenon. However, the natural course of NOAF during long-term follow-up is incompletely understood.

Recent studies suggested that patients with NOAF have a higher risk of ischemic stroke compared to patients with no AF [15–17]. While some studies suggested a higher mortality for NOAF patients, others could not confirm this finding, thus the long-term prognostic implications of NOAF in STEMI are not well-elucidated [18].

AF in patients hospitalized for STEMI represents an important clinical challenge regarding the need for antiarrhythmic therapy and anticoagulation. Previous pilot studies have shown a low rate of adequate anticoagulation therapy in patients with STEMI and AF, possibly related due to concerns of major bleeding [19–21]. This has been identified by the latest guidelines providing now guidance regarding antithrombotic treatment such as the use and duration of triple therapy [22]. The current use of anticoagulation, especially the use of triple therapy in STEMI patients with NOAF, is unknown.

Thus, the purpose of this study was threefold: to assess the incidence of NOAF in patients hospitalized for STEMI, to identify risk factors of AF development, and to analyze its impact on patient care, therapy, and outcomes.

## Methods

### Study population

Consecutive patients who underwent coronary angiography at the University Hospital Basel from May 2015 to September 2023 due to suspected STEMI were included. The diagnosis of a STEMI was based on the third universal definition of myocardial infarction and requires the identification of at least two contiguous leads with ST-segment elevation ≥ 2.5 mm in men < 40 years, or ≥ 2 mm in men ≥ 40 years, or ≥ 1.5 mm in women in leads V_2_-V_3_ and/or ≥ 1 mm in the other leads [23, 24]. Exclusion criteria included patients with a final diagnosis of myopericarditis, coronary spasm, coronary sclerosis without stenosis, subacute aortic dissection, or Takotsubo cardiomyopathy. The study was carried out according to the principles of the Declaration of Helsinki and approved by the local ethics committees.

### Data collection

Available medical records including patient history, physical examination, results of laboratory testing including serial high sensitivity cardiac troponin (hs-cTn) levels, radiologic testing, serial 12-lead ECG testing including findings from telemetric surveillance, echocardiography, lesion severity and morphology in coronary angiography, and discharge summary pertaining to the patient from the time of presentation to the emergency department through long-term follow-up were carefully reviewed. Transthoracic echocardiography (TTE) was performed during the hospital stay by a trained cardiologist or cardiac sonographer and analyzed by a board-certified cardiologist. All STEMI patients were admitted to the intensive care unit with at least 24 h of three-lead ECG monitoring. SB and JM were responsible for collecting and analyzing the data. The follow-up survey took place in September 2023.

### New-onset atrial fibrillation

The occurrence of AF was recorded based on electrocardiograms from patients’ medical records during hospitalization or during follow-up examinations. AF was defined as a cardiac rhythm without detectable repetitive P waves and irregular RR intervals in a standard 12-lead or a single-lead ECG tracing of ≥ 30 s [25]. Patients were classified according to the time at which AF became clinically apparent. Patients who were in sinus rhythm on admission and developed AF at any time during the initial hospitalization or in 12-month follow-up were classified as NOAF. In addition, we performed a sensitivity analysis only including patients with NOAF during the initial hospitalization. Patients with a diagnosis of AF prior to the STEMI admission were considered patients with prior AF.

### Outcomes

The primary study outcome was the incidence of NOAF in STEMI patients. The secondary study outcome was the occurrence of major adverse cardiac events (MACE) including stroke, major bleeding, and death during long-term follow-up of NOAF in STEMI patients. For strokes, both hemorrhagic and ischemic events were considered. Major bleeding was defined according to the recommended ISTH criteria [26].

### Statistical analysis

Continuous variables were presented as mean ± SD or median with interquartile range, and non-normally distributed data was analyzed using the Mann–Whitney *U* test or Kruskal–Wallis rank sum as appropriate. Normality was assessed visually using histograms. Categorical variables were expressed as numbers and percentages and compared using the chi-squared test or Fisher’s exact test as appropriate. A *p*-value < 0.05 was considered statistically significant. Kaplan–Meier estimates with a log-rank test were carried out for the secondary endpoints and presented graphically. Potential risk factors for NOAF and AF within 12 months were first tested in a univariable logistic regression. Variables with a *p* < 0.05 in the univariable analysis were then included in a multivariable model. Data was analyzed, and graphs were created using R version 4.3.2 (R Foundation 180 for Statistical Computing, Vienna, Austria) with RStudio (version 2023.09.1) [27].

## Results

### Study population

A total of 1358 patients underwent coronary angiography at the University Hospital Basel from May 2015 to September 2023 due to suspected STEMI. After excluding patients with ST-segment elevation but a final diagnosis of myopericarditis (*n* = 9), coronary sclerosis without stenosis (*n* = 8), coronary spasm (*n* = 8), subacute aortic dissection or aneurysm (*n* = 2), coronary artery dissection (*n* = 2), or Takotsubo cardiomyopathy (*n* = 28), 1301 patients were eligible for analysis. A patient flow chart is provided in Supplemental Figure S1.

### New-onset atrial fibrillation (NOAF)

**Fig. 1:**
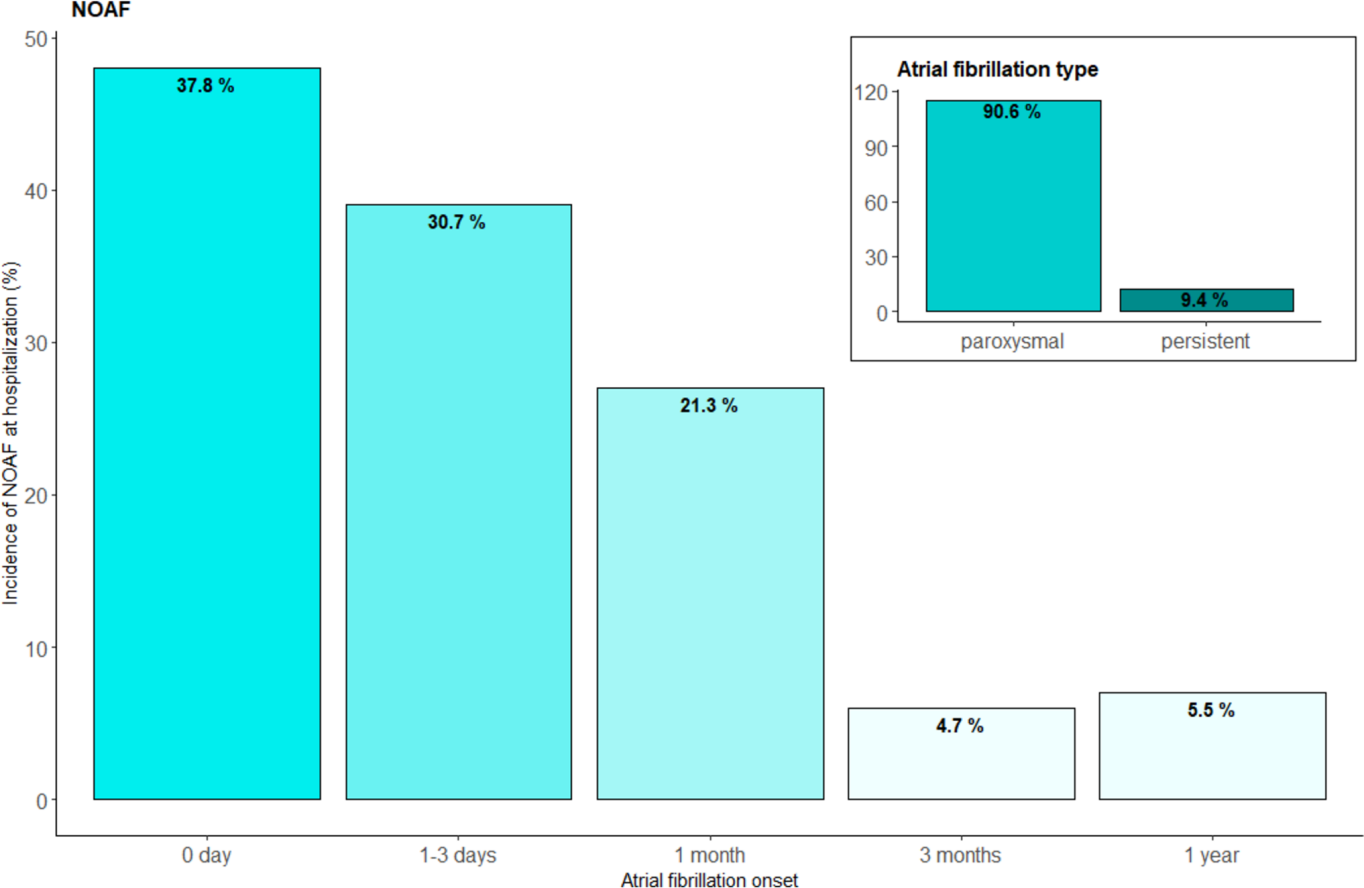
Onset of new-onset atrial fibrillation: The timepoint of new-onset atrial fibrillation is visualized as a bar plot. In the right upper corner, the type of new-onset atrial fibrillation is shown. NOAF new-onset atrial fibrillation

AF was recorded in 17% of all patients (*n* = 222). Among these, 58% (*n* = 128) had NOAF and 31% (*n* = 68) had pre-existing AF prior to the STEMI admission. Of the 128 patients with NOAF, 38% (*n* = 48) were established with AF in the cardiac catheter laboratory, 31% (*n* = 39) during days 1–3, and in 32% > day 3 (*n* = 41). One hundred fifteen (91%) patients suffered from paroxysmal AF and 12 (9.4%) patients from persistent AF (Fig. 1).

### Baseline characteristics

Baseline characteristics are shown in Table 1. The mean age was 63 ± 12, 21% were female, median pain duration was 3 h, and 5.9% were in cardiogenic shock. When comparing patients with NOAF vs. patients with no AF, patients were significantly older with a mean age of 69 ± 11 vs. 63 ± 12, *p* < 0.001, were more often in cardiogenic shock with 11% vs. 5.2%, *p* = 0.038, and showed significantly higher GRACE scores with 178 [160–207] vs. 156 [137–177], *p* < 0.001, respectively. Baseline characteristics stratified for patients with NOAF and with prior AF are summarized in Supplemental Table S1, where NOAF vs. prior AF patients differed only in age (69 [± 11] vs. 74 [± 9] years, *p* = 0.013).

**Table 1:** Baseline characteristics

	Overall, *N* = 1207^a^	AF status		*p*-value^c^
		NOAF, *N* = 128^b^	No AF, *N* = 1079^b^	
Age (years)	63 [12]	69 [11]	63 [12]	** < 0.001**
Female	258 (21%)	33 (26%)	225 (21%)	0.21
HR (bpm)	75 [65–84]	78 [70–90]	75 [65–84]	**0.020**
Systolic BP (mmHg)	117 [107–130]	118 [102–131]	117 [107–130]	0.61
Diastolic BP (mmHg)	73 [64–81]	72 [60–81]	73 [64–80]	0.44
Weight (kg)	80 [72–90]	80 [72–85]	80 [72–90]	0.47
BMI (kg/m^2^)	26 [24–29]	27 [25–30]	26 [24–29]	0.41
Typical Angina pectoris (*N* = 1002)	944 (94%)	83 (89%)	861 (95%)	0.057
Pain duration (h)	3 [2–6]	3 [2–10]	3 [2–6]	0.67
Cardiogenic shock (*N* = 901)	53 (5.9%)	11 (11%)	42 (5.2%)	**0.038**
Hypertension (*N* = 489)	408 (83%)	44 (83%)	364 (83%)	0.93
Diabetes (*N* = 124)				0.093
IDDM	27 (22%)	6 (40%)	21 (19%)	
NIDDM	97 (78%)	9 (60%)	88 (81%)	
GRACE score	157 [139–181]	178 [160–207]	156 [137–177]	** < 0.001**

^a^*n*/*N* (%); mean [SD]; median [IQR]

^b^Mean (SD), median [IQR], or frequency (%)

^c^Pearson’s chi-squared test; Wilcoxon rank sum test; Fisher’s exact test

*GRACE* Global Registry of Acute Coronary Events, *IDDM* insulin-dependent diabetes mellitus, *NIDDM* non-insulin-dependent diabetes mellitus

### Transthoracic echocardiography findings

Echocardiographic measurements are summarized in Table 2. Patients with NOAF demonstrated lower left ventricular ejection fraction (LVEF) compared to patients with no AF, 45% vs. 50%, *p* < 0.001, respectively. The left and right atrial diameters were significantly increased in patients with NOAF compared to patients with no AF: left atrial volume index (LAVI) 35 [28–41] ml/m^2^ vs. 27 [22–34] ml/m^2^, *p* < 0.001, and right atrial area (RA) 17 [14–20] cm^2^ vs. 15 [13–17] cm^2^, *p* = 0.016, respectively.

**Table 2:** Echocardiographic parameters

	Overall, *N* = 1207^a^	AF status		*p*-value ^c^
		NOAF, *N* = 128^b^	No AF, *N* = 1079^b^	
Left atrial volume [ml]	53 [42–68]	68 [53–81]	52 [41–66]	** < 0.001**
Left atrial volume index [ml/m^2^]	28 [22–35]	35 [28–41]	27 [22–34]	** < 0.001**
Left ventricular end-diastolic diameter [mm]	47 [43–51]	47 [43–51]	46 [43–51]	0.43
Left ventricular end-systolic diameter [mm]	32 [28–36]	33 [29–38]	32 [28–36]	**0.038**
Left ventricular ejection fraction (%)	50 [42–56]	45 [39–54]	50 [44–57]	** < 0.001**
Left atrial diameter [mm]	36 [33–40]	39 [33–42]	36 [33–40]	** < 0.001**
Right atrial area [cm^2^]	15 [13–18]	17 [14–20]	15 [13–17]	**0.016**
End diastolic volume[ml]	99 [79–121]	102 [79–125]	99 [79–120]	0.36
End diastolic volume index [ml/m^2^]	51 [42–61]	52 [40–65]	51 [42–61]	0.63
Mitral insufficiency (*N* = 781)				0.065
Mild	698 (89%)	89 (85%)	609 (90%)	
Moderate	75 (9.6%)	13 (12%)	62 (9.2%)	
Severe	8 (1.0%)	3 (2.9%)	5 (0.7%)	

^a^Median [IQR]; *n*/*N* (%)

^b^Mean (SD), median [IQR], or frequency (%)

^c^Wilcoxon’s rank sum test; Fisher’s exact test

### Coronary angiogram findings

Coronary angiogram findings are summarized in Table 3. The culprit vessel was the right coronary artery (RCA) in 42 patients (33%), the left circumflex artery (LCX) in 17 patients (13%), and the left anterior descending artery (LAD) in 27 patients (21%) in patients with NOAF. There was no difference to patients with no AF. Patients with NOAF significantly less often suffered single-vessel disease compared to patients with no AF with 29% vs. 40%, *p* = 0.026, and significantly more often suffered triple-vessel disease with 37% vs. 29%, *p* = 0.044. The left anterior descending artery (LAD) was most frequently affected in patients with NOAF with stroke (*n* = 7, 54%) and the right coronary artery (RCA) in patients with major bleeding (*n* = 5, 56%).

**Table 3:** Affected vessel in coronary angiography

	Overall, *N* = 1207^a^	AF status		*p*-value^c^
		NOAF, *N* = 128^b^	No AF, *N* = 1079^b^	
100% stenoses				
RCA	350 (29%)	42 (33%)	308 (29%)	0.31
LCX	159 (13%)	17 (13%)	142 (13%)	0.97
LAD	298 (25%)	27 (21%)	271 (25%)	0.32
Vessel disease				
Single-vessel	461 (38%)	37 (29%)	424 (40%)	**0.026**
Dual-vessel	383 (32%)	42 (33%)	341 (32%)	0.73
Triple-vessel	354 (30%)	47 (37%)	307 (29%)	**0.044**

^a^*n*/*N* (%)

^b^Mean (SD), median [IQR], or frequency (%)

^c^Pearson’s chi-squared test

*RCA* right coronary artery, *LCX* left circumflex artery, *LAD* left anterior descending artery

### Anticoagulation

The choice of anticoagulation therapy after the index procedure is summarized in Table 4. Among patients with NOAF, 60 patients (47%) received triple therapy for 1 week thereafter switching to dual therapy, 55 patients (43%) dual antiplatelet therapy, 9 patients (7%) oral anticoagulation (OAC) and single platelet therapy, 2 patients (1.6%) single OAC, and 2 patients (1.6%) single platelet therapy. When comparing NOAF patients with patients with prior AF, patients with NOAF significantly less often received triple therapy (47% vs. 66%, *p* = 0.010) and significantly more often received dual antiplatelet therapy (43% vs. 15%, *p* < 0.001) (Supplemental Table S2).

**Table 4:** Anticoagulation after STEMI

	Overall, *N* = 1207^a^	AF status		*p*-value^c^
		NOAF, *N* = 128^b^	No AF, *N* = 1079^b^	
OAC only	2 (0.2%)	2 (1.6%)	0 (0%)	**0.012**
Single antiplatelet	9 (0.8%)	2 (1.6%)	7 (0.7%)	0.25
OAC and single antiplatelet	17 (1.4%)	9 (7.0%)	8 (0.8%)	** < 0.001**
Dual antiplatelet	1058 (89%)	55 (43%)	1003 (95%)	** < 0.001**
Triple therapy: OAK and dual antiplatelet	100 (8.4%)	60 (47%)	40 (3.8%)	** < 0.001**

^a^*n*/*N* (%)

^b^Mean (SD), median [IQR], or frequency (%)

^c^Pearson’s chi-squared test; Fisher’s exact test

*OAC* oral anticoagulants

### Outcomes

During a median follow-up of 683 days, in patients with NOAF compared to patients with no AF, stroke occurred in 13 patients (10%) vs. 41 patients (3.8%), *p* = 0.001, major bleeding was found in 9 patients (7%) vs. 18 patients (1.7%), *p* = 0.001, and death was reported in 21 patients (16%) vs. 73 patients (6.8%), *p* < 0.001, respectively. When comparing patients with NOAF to patients with prior AF, strokes occurred in 13 patients (10%) vs. 4 (5.9%) (*p* = 0.31), major bleeding in 9 patients (7%) vs. 5 patients (7.4%) (*p* > 0.99), and deaths in 21 patients (16%) vs. 15 patients (22%) (*p* = 0.33).

**Fig. 2:**
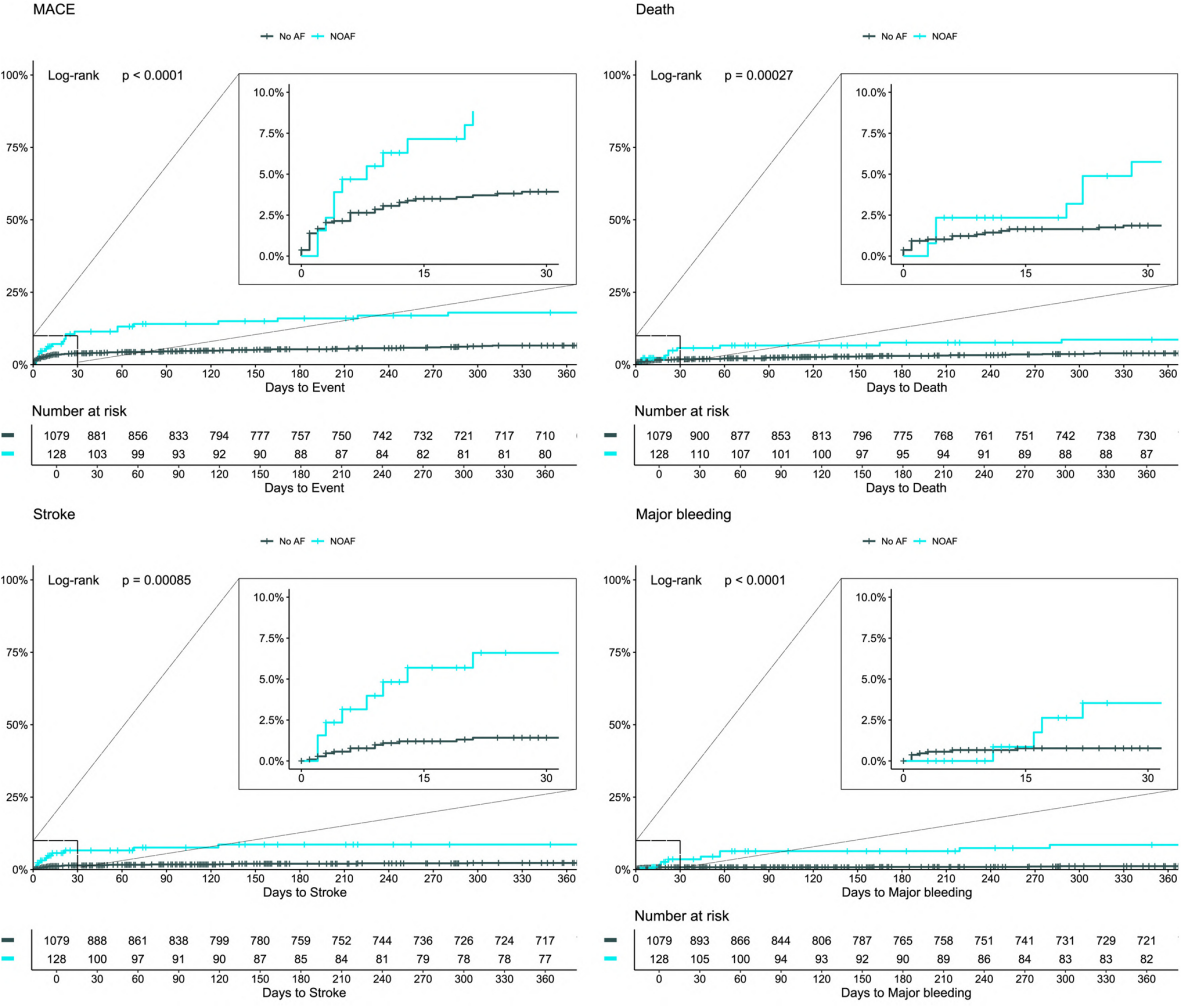
Kaplan–Meier plots of MACE, death, stroke, and major bleeding: Secondary endpoints during 365 days and in an extended frame during the first 30 days after STEMI for NOAF (blue) and no AF (black). AF atrial fibrillation, MACE major adverse cardiac events, NOAF new-onset atrial fibrillation

Kaplan–Meier curves are presented in Fig. 2. When comparing patients with NOAF and patients with no AF, a significant difference was found for in-hospital outcomes, outcomes for 12 months follow-up, and long-term follow-up for all MACE (*p* < 0.0001) and for each secondary outcome separately: for stroke (*p* = 0.00085), major bleeding (*p* < 0.0001), and death (*p* = 0.00027). Supplemental Tables S3 to S5 contain a detailed overview of clinical characteristics of patients with NOAF suffering from a stroke, major bleeding, or death.

Findings were confirmed when only including patients with NOAF occurring during the index-hospitalization (*n* = 110) (Supplemental Figure S2).

### Risk factors of NOAF

A multivariable regression model using variables from baseline patient characteristics identified by univariable analysis showed that age (OR 1.05, 95% CI 1.02–1.07, *p* = 0.001), LVEF (OR 0.96, 95% CI 0.95–0.98, *p* = 0.001), and left atrial diameter (OR 1.08, 95% CI 1.03–1.13, *p* = 0.002) are independent risk factors for the occurrence of NOAF in STEMI patients (Table 5).

**Table 5:** Univariable and multivariable logistic regression results for risk factors of NOAF

Variables	Univariable model		Multivariable model^a^	
	OR (95% CI)	*p*-value^b^	Adjusted OR (95% CI)	*p*-value^b^
Patient characteristics				
Age, y (per unit increase)	1.05 (1.03–1.06)	** < 0.001**	1.05 (1.02–1.07)	**0.001**
Sex (female)	1.32 (0.86–2.01)	0.20	-	-
BMI	1.02 (0.96–1.09)	0.47	-	-
Transthoracic echocardiography				
LVEF	0.96 (0.95–0.98)	** < 0.001**	0.96 (0.93–0.98)	**0.001**
LA diameter	1.09 (1.05–1.14)	** < 0.001**	1.08 (1.03–1.13)	**0.002**
Score				
GRACE score	1.02 (1.01–1.02)	** < 0.001**	1.01 (1.00–1.02)	0.205
Vessel disease				
Single-vessel	0.64 (0.42–0.94)	**0.027**	1.04 (0.56–1.95)	0.893
Dual-vessel	1.07 (0.72–1.58)	0.73	-	
Triple-vessel	1.48 (1.00–2.17)	**0.045**	0.92 (0.49–1.73)	0.790

^a^Multivariable model includes variables with *p* < 0.05 on univariable logistic regression

^b^Pearson’s chi-squared test

*CI* confidence interval, *LA* left atrial, *LVEF* left ventricular ejection fraction [%], *OR* odds ratio

### Risk factors of MACE among all AF patients and NOAF patients

Among patients with NOAF, in the univariable model, age (OR 1.06, 95% CI 1.02–1.11, *p* = 0.004) and GRACE score (OR 1.02, 95% CI 1.01–1.03, *p* = 0.010) were risk factors for MACE, while in the multivariable model, only the GRACE score was an independent risk factor with an OR 1.02 (95% CI 1.00–1.03, *p* = 0.030, Table 6). Univariable and multivariable logistic regression results for risk factors of MACE in all AF patients are presented in Supplemental Table S6.

**Table 6:** Univariable and multivariable logistic regression results for risk factors of MACE in NOAF (*n* = 128)

Variables	Univariable model		Multivariable model^a^	
	OR (95% CI)	*p*-value^b^	Adjusted OR (95% CI)	*p*-value^b^
Patient characteristics				
Age, y (per unit increase)	1.06 (1.02–1.11)	**0.004**	1.04 (0.99–1.09)	0.097
Sex (female)	1.66 (0.68–3.91)	0.25	-	-
BMI	0.90 (0.76–1.04)	0.18	-	-
Transthoracic echocardiography				
LVEF	0.98 (0.94–1.02)	0.34	-	-
LA diameter	1.04 (0.96–1.13)	0.37	-	-
Score				
GRACE score	1.02 (1.01–1.03)	**0.010**	1.02 (1.00–1.03)	**0.030**
Vessel disease				
Single-vessel	0.87 (0.35–2.06	0.76	-	-
Dual-vessel	0.56 (0.21–1.32)	0.20	-	
Triple-vessel	1.88 (0.84–4.24)	0.12	-	-
Anticoagulation				
Dual	0.92 (0.41–2.04)	0.84		
Triple	0.93 (0.41–2.05)	0.85		

^a^Multivariable model includes variables with *p* < 0.05 on univariable logistic regression

^b^Pearson’s chi-squared test

*CI* confidence interval, *LA* left atrial, *LVEF* left ventricular ejection fraction [%], *OR* odds ratio

## Discussion

In this large cohort of STEMI patients, our aim was to assess the incidence of NOAF, to identify risk factors of AF development, and to analyze its impact on patient care, therapy, and outcomes during long-term follow-up.

We report the following main findings: First, the incidence of NOAF was 9.8% with 68% of AF cases occurring during the first 3 days of hospitalization. Second, patients with NOAF were older, were more often in cardiogenic shock, and showed significantly higher GRACE scores. Baseline characteristic between patients with NOAF and a prior history of AF also differed in terms of age, with patients with NOAF being younger. Third, patient with NOAF demonstrated increased left and right atrial diameters. Fourth, patients with NOAF significantly more often suffered triple-vessel disease with 37% vs. 29%, *p* = 0.024. Interestingly, the LAD was most frequently affected in patients with NOAF with stroke (54%) and the RCA in patients with major bleeding (56%). Fifth, most importantly, among patients with NOAF, less than half of patients (47%) received triple therapy as recommended by current guidelines. Sixth, during a median follow-up of 683 days, in patients with NOAF compared to patients with no AF, a significant difference was found for in-hospital outcomes, outcomes for 12-month follow-up, and long-term follow-up for stroke, major bleeding, and death. Seventh, a multivariable regression showed that age, LVEF, and LA diameter were independent risk factors for the occurrence of NOAF.

These findings corroborate and extend findings from previous studies. The incidence of NOAF of 9.8% is consisting with the most recent literature: Lin et al. found a comparable incidence of NOAF of 11% in 783 STEMI patients [28], and Siu et al. [29] reported an incidence of NOAF of 13% (*n* = 431). The latter study found a higher incidence of NOAF in patients with inferior STEMI [29]. A lower incidence of NOAF in STEMI was noted by Mrdovic et al. with 6.2% (*n* = 2096), even though patients with a prior history of paroxysmal AF were classified as NOAF [18]. Similarly to this study, an older population of patients suffering from NOAF compared to those without AF has been described [15, 30].

The use of triple therapy in NOAF STEMI patients is in line with a previous pilot study. Hofer et al. reported that triple therapy has been significantly less frequently used in NOAF patients compared to patients with prior AF (38% vs. 66%) [31]. AF in the setting of STEMI is associated with poor outcomes [8]. Previous studies showed for AMI patients with AF a higher risk of short-term as well as long-term mortality, stroke, and bleeding [15–17]. Similarly to this study, Obayashi et al. found that in patients with NOAF, the long-term risk of mortality was comparable to that of patients with prior AF and significantly higher than in patients with no AF [32]. It remains uncertain whether NOAF independently contributes to these outcomes or merely serves as an indicator of disease severity and poor prognosis [8]. Regarding risk factors, age seems to be confirmed to be an independent risk factor of NOAF in several previous studies [9, 29, 30]. Galvão et al. also found decreased LVEF and LA diameter to be independent risk factors of NOAF [33].

While the results of this study align with previous findings regarding NOAF in STEMI patients, they also highlight several aspects that could influence clinical practice moving forward: there is currently a lack of a standardized approach to anticoagulation in STEMI patients with NOAF, particularly those with elevated CHA2DS2-VASc scores. It is important to note that the risk of major bleeding is also high in these patients. There is a lack of data and thus consensus on how to best balance these risks in this challenging population. The high incidence of NOAF occurring within the first few days of hospitalization indicates the importance of vigilant monitoring during this period. Identifying independent risk factors for NOAF could enhance risk stratification in clinical practice by targeting higher-risk patients for closer monitoring. Lastly, the lack of a measurable effect in this cohort on bleeding rates and on stroke events when comparing the use of a dual vs. a triple anticoagulation strategy highlights the need for further research in this field, especially regarding the optimal anticoagulation therapy in this challenging patient population.

We acknowledge the following limitations: First, this was a single-center study with all its accompanying limitations. Second, we did not assess the association of developing AF with obstructive coronary disease inside or proximal to the nodal arteries. A prior pilot study showed that a higher burden of coronary artery disease within all arteries supplying blood flow to the atrial myocardium was associated with higher odds of NOAF at 1 year [34]. Third, we were not able to assess the AF burden of NOAF patients. It is currently unclear whether NOAF in STEMI patients can be regarded as an isolated event or predicts subsequent AF episodes long term. There is increasing evidence that AF burden can predict adverse outcomes such as heart failure, cerebrovascular events, and mortality [35]. Future studies assessing AF burden short-term, for example, by using wearable devices such as smartwatches or ECG patches, are warranted. This data would also provide guidance regarding the appropriate use of immediate restoration of sinus rhythm by pharmacologic or electric cardioversion. Similarly, to patients with recent-onset AF, a wait-and-see approach might also be non-inferior in the NOAF-STEMI population [36]. Fourth, we did not assess the impact of the currently proposed CHADS-VA score [37]. Fifth, there is increasing evidence that current ST elevation criteria are not a good surrogate for occlusive myocardial infarction [38, 39].

## Conclusion

In conclusion, almost 1 in 10 STEMI patients were found to have NOAF. Patients with NOAF in the context of STEMI had worse outcomes during hospitalization and a less favorable prognosis during long-term follow-up compared to patients without AF. In addition to age, lower LVEF and larger LA diameter are independent risk factors for NOAF. Future studies assessing optimal anticoagulation therapy in this challenging patient population are warranted.

## Supplementary Information

Below is the link to the electronic supplementary material.

Supplementary file1 (DOCX 3084 KB)

## Data Availability

Data are available from the authors upon request.

## Abbreviations

AF: Atrial fibrillation
AMI: Acute myocardial infarction
CAG: Coronary angiography
GRACE: Global Registry of Acute Coronary Events
hs-cTn: High sensitivity cardiac troponin
ISTH: International Society on Thrombosis and Haemostasis
LA: Left atrium
LAD: Left anterior descending artery
LAVI: Left atrial volume index
LCX: Left circumflex artery
LVEF: Left ventricular ejection fraction
MACE: Major adverse cardiac events
NOAF: New-onset atrial fibrillation
OAC: Oral anticoagulants
RA: Right atrial area
RCA: The right coronary artery
STEMI: ST-segment elevation myocardial infarction
TTE: Transthoracic echocardiography

## Declarations

Philipp Krisai reports speaker fees from BMS/Pfizer. Grants from the Swiss National Science Foundation, Swiss Heart Foundation, Foundation for Cardiovascular Research Basel, and Machaon Foundation.

Thomas Nestelberger has received research support from the Swiss National Science Foundation (P400PM_191037/1), the Prof. Dr. Max Cloëtta Foundation, the Margarete und Walter Lichtenstein-Stiftung (3MS1038), and the University Hospital Basel as well as speaker/consulting honoraria or research support from Edwards Lifesciences, Pronova Medical, Boston Scientific, Medtronic, Abbott, Beckman Coulter, Bayer, Ortho Clinical Diagnostics and Orion Pharma, outside the submitted work.

Jasper Boeddinghaus is supported by an Edinburgh Doctoral College Scholarship and received research grants from the University of Basel, the University Hospital of Basel, the Division of Internal Medicine, the Swiss Academy of Medical Sciences, the Gottfried and Julia Bangerter-Rhyner Foundation, the Swiss National Science Foundation, and the Freie Akademische Gesellschaft Basel as well as honoraria from Siemens, Roche Diagnostics, Ortho Clinical Diagnostics, Quidel Corporation, Beckman Coulter, and travel support from Medtronic, all outside the submitted work.

Christian Sticherling: Member of Medtronic Advisory Board Europe and Boston Scientific Advisory Board Europe, received educational grants from Biosense Webster and Biotronik and a research grant from the European Union’s FP7 program and Biosense Webster and lecture and consulting fees from Abbott, Medtronic, Biosense-Webster, Boston Scientific, Microport, and Biotronik all outside the submitted work.

Michael Kühne reports grants from Bayer, grants from BMS, grants from Boston Scientific, grants from Daiichi Sankyo, grants from Pfizer, personal fees from Abbott, personal fees from Boston Scientific, personal fees from Daiichi Sankyo, grants from the Swiss National Science Foundation (Grant numbers 33CS30_148474, 33CS30_177520, 32473B_176178), the Swiss Heart Foundation, the Foundation for Cardiovascular Research Basel, and the University of Basel. Royalties from Springer (ECG book), all outside the submitted work.

Dr. Mueller has received research support from the Swiss National Science Foundation, the Swiss Heart Foundation, the University of Basel, the University Hospital Basel, Abbott, Astra Zeneca, Beckman Coulter, Boehringer Ingelheim, Idorsia, LSI-Medience, Novartis, Ortho Clinical Diagnostics, Quidel, Roche, Siemens, Singulex, Sphingotec, and SpinChip, as well as speaker honoraria/consulting honoraria from Acon, Amgen, Astra Zeneca, Boehringer Ingelheim, Bayer, BMS, Idorsia, Novartis, Osler, Roche, Sanofi, and SpinChip, outside of the submitted work, and all paid to the institution.

The remaining authors have nothing to declare.
