# Supplementary material for: Impact of new-onset atrial fibrillation in patients with ST-segment elevation myocardial infarction
Source: J Interv Card Electrophysiol. 2024 Dec 11;68(3):655–65. doi: 10.1007/s10840-024-01941-5 (PMC12167322; doi:10.1007/s10840-024-01941-5)
Supplement: Supplementary file 1 — Supplementary file1 (DOCX 3084 KB) [file 10840_2024_1941_MOESM1_ESM.docx]

## Supplementary Information

### Supplemental Figure S1 Patient flow chart


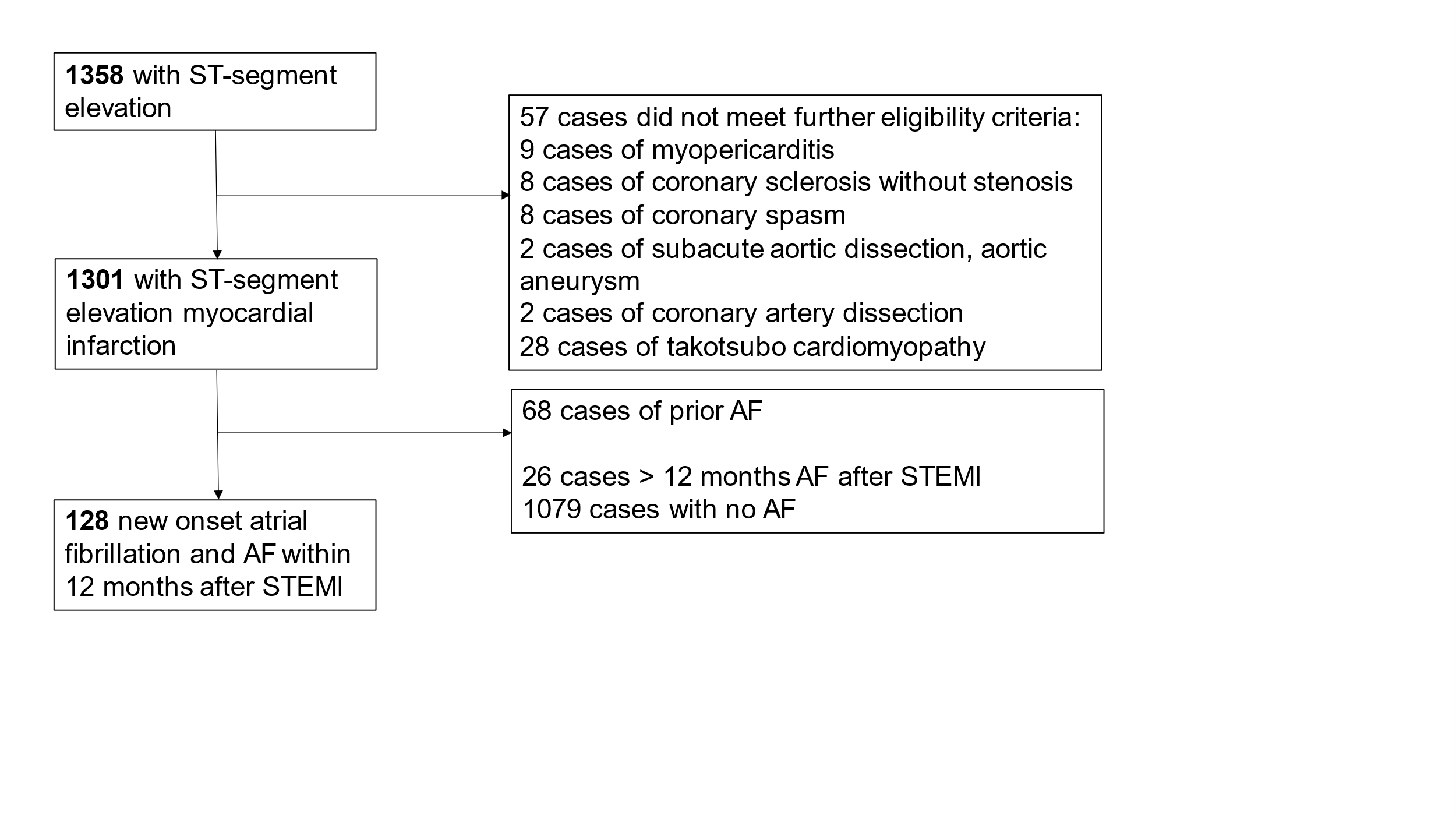


### Supplemental Figure S2: Kaplan Meier plots of MACE, Death, Stroke and Major bleeding of the subgroup NOAF occurring during the index-hospitalization (n=110)


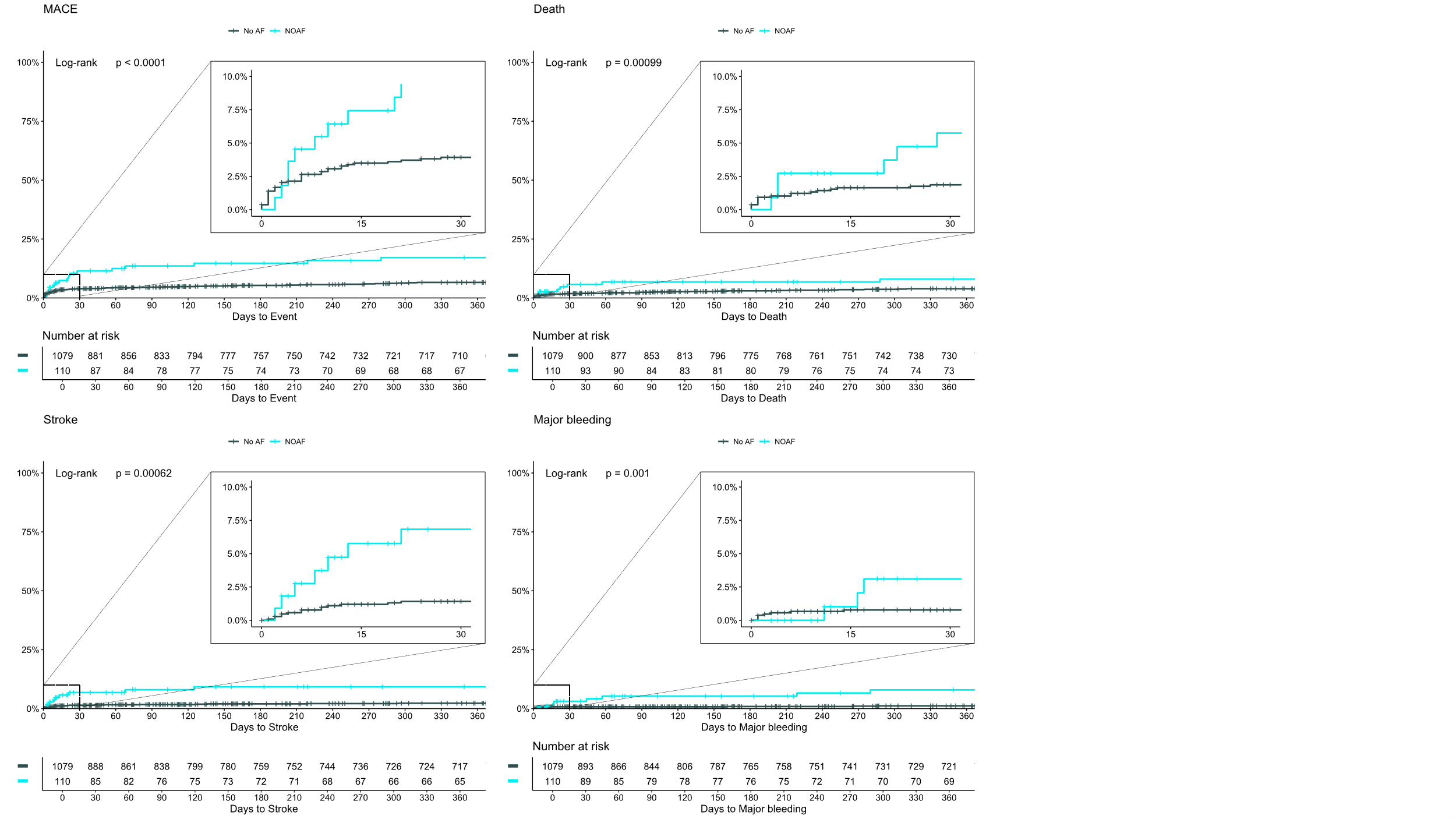


### Table S1 Baseline characteristics NOAF versus Prior AF

|  |  | **Atrial fibrillation status** | | |  |
| --- | --- | --- | --- | --- | --- |
|  | **Overall,**  N = 196^a^ | **NOAF,**  N = 128^b^ | **Prior AF,**  N = 68^b^ | **p-value**^c^ |  |
| **Age (years)** | 71 [11] | 69 [11] | 74 [9] | **0.013** |  |
| **Female** | 47 (24%) | 33 (26%) | 14 (21%) | 0.48 |  |
| **HR (bpm)** | 77 [67-87] | 78 [70-90] | 75 [66-83] | 0.14 |  |
| **Systolic BP (mmHg)** | 118 [105-133] | 118 [102-131] | 117 [107-135] | 0.43 |  |
| **Diastolic BP (mmHg)** | 72 [60-81] | 72 [60-81] | 74 [61-83] | 0.70 |  |
| **Weight (kg)** | 80 [73-85] | 80 [72-85] | 79 [74-89] | 0.86 |  |
| **BMI (kg/m^2^)** | 26 [25-31] | 27 [25-30] | 26 [24-32] | 0.80 |  |
| **Typical Angina pectoris**  (N= 143) | 127 (89%) | 83 (89%) | 44 (88%) | 0.79 |  |
| **Pain duration (h)** | 3 [2-8] | 3 [2-10] | 3 [2-4] | 0.76 |  |
| **Cardiogenic Shock**  (N= 148) | 17 (11%) | 11 (11%) | 6 (13%) | 0.79 |  |
| **Hypertension**  (N= 79) | 68 (86%) | 44 (83%) | 24 (92%) | 0.32 |  |
| **Diabetes**  (N= 26) |  |  |  | 0.39 |  |
| IDDM | 8 (31%) | 6 (40%) | 2 (18%) |  |  |
| NIDDM | 18 (69%) | 9 (60%) | 9 (82%) |  |  |
| **GRACE Score** | 182 [164-209] | 178 [160-207] | 186 [167-209] | 0.17 |  |
| ^a^n / N (%); Median [IQR] | | | | | |
| ^b^Mean (SD), Median [IQR] or Frequency (%) | | | | | |
| ^c^Wilcoxon rank sum test; Fisher's exact test; Pearson's Chi-squared test  GRACE: global registry of acute coronary events, IDDM: insulin dependent diabetes mellitus, NIDDM: non-insulin dependent diabetes mellitus | | | | | |

### Table S2 Anticoagulation after STEMI

|  |  | **AF status** | |  |
| --- | --- | --- | --- | --- |
|  | **Overall**,  N = 196^a^ | **NOAF**,  N = 128^2^ | **Prior AF**,  N = 68^b^ | **p-value**^c^ |
| **OAC only** | 5 (2.6%) | 2 (1.6%) | 3 (4.4%) | 0.34 |
| **Single antiplatelet** | 4 (2.0%) | 2 (1.6%) | 2 (2.9%) | 0.61 |
| **OAC and single antiplatelet** | 17 (8.7%) | 9 (7.0%) | 8 (12%) | 0.29 |
| **Dual antiplatelet** | 65 (33%) | 55 (43%) | 10 (15%) | **<0.001** |
| **Triple therapy: OAK and dual antiplatelet** | 105 (54%) | 60 (47%) | 45 (66%) | **0.010** |
| ^a^n / N (%) | | | | |
| ^b^Mean (SD), Median [IQR] or Frequency (%) | | | | |
| ^c^Pearson's Chi-squared test; Fisher's exact test  OAC: oral anticoagulants | | | | |

### Table S3 Patients (NOAF) with STEMI and Stroke (n = 13)

| Age | Sex | STEMI date | Stenosed vessel | Triple | Anticoagulation after STEMI | Stroke date | Days after STEMI | CHA2DS2VASc Score |
| --- | --- | --- | --- | --- | --- | --- | --- | --- |
| 76 | m | 11.10.2020 | LAD mid | yes | OAC + dual antiplatelet | 13.10.2020 | 2 | 6 |
| 62 | f | 24.09.2020 | RCA prox | yes | OAC + dual antiplatelet | 07.10.2020 | 13 | 8 |
| 84 | f | 12.12.2020 | LAD prox | yes | OAC + dual antiplatelet | 30.05.2023 | 899 | 5 |
| 78 | m | 28.03.2016 | LCA | no | OAC + single antiplatelet | 05.04.2016 | 8 | 7 |
| 82 | f | 27.02.2022 | LAD apical | no | dual antiplatelet | 29.03.2022 | 2 | 5 |
| 77 | m | 22.06.2015 | LAD prox | no | OAC + single antiplatelet | 08.08.2021 | 2239 | 8 |
| 79 | m | 09.06.2023 | LCX distal | no | dual antiplatelet | 16.08.2023 | 68 | 5 |
| 77 | f | 08.12.2015 | RCA PD | no | dual antiplatelet | 11.04.2016 | 125 | 3 |
| 73 | m | 20.05.2019 | LAD prox | no | dual antiplatelet | 23.05.2019 | 3 | 6 |
| 49 | m | 22.04.2019 | LAD prox | yes | OAC + dual antiplatelet | 13.05.2019 | 21 | 2 |
| 78 | f | 01.10.2018 | RCA distal | yes | OAC + dual antiplatelet | 03.06.2023 | 1706 | 7 |
| 72 | m | 02.03.2019 | LAD prox | no | single antiplatelet | 12.03.2019 | 10 | 4 |
| 74 | f | 30.03.2019 | RCA distal | yes | OAC + dual antiplatelet | 04.04.2019 | 5 | 6 |

RCA: right coronary artery, LCX: left circumflex artery, LAD: left anterior descending artery

### Table S4 Patients (NOAF) with STEMI and Major bleeding (n = 9)

| Age | Sex | STEMI date | Stenosed vessel | Triple | Anticoagulation after STEMI | Major bleeding date | Days after STEMI | HAS-BLED Score |
| --- | --- | --- | --- | --- | --- | --- | --- | --- |
| 86 | m | 30.09.2020 | RCA prox | no | dual antiplatelet | 30.09.2020 | 0 | 5 |
| 68 | m | 04.08.2020 | LCX prox | no | OAC + single antiplatelet | 30.09.2020 | 57 | 3 |
| 47 | f | 30.01.2023 | RCA prox | no | dual antiplatelet | 30.01.2023 | 0 | 0 |
| 92 | f | 10.01.2021 | RCA prox | yes | OAC + dual antiplatelet | 17.10.2021 | 280 | 4 |
| 75 | m | 31.05.2023 | LAD mid | no | dual antiplatelet | 27.07.2023 | 57 | 3 |
| 77 | f | 08.12.2015 | RCA PD | no | dual antiplatelet | 21.01.2016 | 44 | 2 |
| 61 | m | 06.09.2019 | RCA mid | yes | OAC + dual antiplatelet | 28.09.2019 | 22 | 2 |
| 73 | m | 20.05.2019 | LAD prox | no | dual antiplatelet | 06.06.2019 | 17 | 2 |
| 72 | m | 02.03.2019 | LAD prox | no | single antiplatelet | 13.03.2019 | 11 | 3 |

RCA: right coronary artery, LCX: left circumflex artery, LAD: left anterior descending artery

### Table S5 Patients (NOAF) with STEMI and Death (n = 21)

| Age | Sex | STEMI date | Stenosed vessel | Triple | Anticoagulation after STEMI | Date of death | Days after STEMI |
| --- | --- | --- | --- | --- | --- | --- | --- |
| 86 | m | 30.09.2020 | RCA prox | no | dual antiplatelet | 21.10.2021 | 386 |
| 76 | f | 06.08.2015 | RCA RV | yes | OAC + dual antiplatelet | 25.09.2020 | 1877 |
| 68 | m | 04.08.2020 | LCX prox | no | OAC + single antiplatelet | 30.09.2020 | 57 |
| 66 | m | 12.01.2016 | LCX distal | no | OAC + single antiplatelet | 03.02.2016 | 22 |
| 76 | m | 11.10.2020 | LAD mid | yes | OAC + dual antiplatelet | 14.10.2020 | 3 |
| 84 | f | 12.12.2020 | LAD prox | yes | OAC + dual antiplatelet | 10.06.2023 | 910 |
| 92 | f | 10.01.2021 | RCA prox | yes | OAC + dual antiplatelet | 25.10.2021 | 288 |
| 70 | m | 08.03.2020 | RCA distal | yes | OAC + dual antiplatelet | 28.03.2020 | 20 |
| 83 | m | 30.12.2020 | RCA prox | no | dual antiplatelet | 13.07.2022 | 560 |
| 78 | m | 28.03.2016 | LCA | no | OAC + single antiplatelet | 13.08.2023 | 2694 |
| 59 | m | 31.05.2017 | RCA prox | no | dual antiplatelet | 12.11.2017 | 165 |
| 92 | m | 04.11.2016 | RCA prox | yes | OAC + dual antiplatelet | 18.08.2023 | 2478 |
| 89 | m | 30.06.2015 | LAD prox | no | dual antiplatelet | 14.03.2023 | 2814 |
| 61 | m | 12.04.2017 | LCA | yes | OAC + dual antiplatelet | 26.07.2020 | 1201 |
| 61 | m | 06.09.2019 | RCA mid | yes | OAC + dual antiplatelet | 28.09.2019 | 22 |
| 76 | f | 26.03.2019 | RCA prox | yes | OAC + dual antiplatelet | 23.04.2019 | 28 |
| 88 | m | 30.05.2018 | RCA mid | yes | OAC + dual antiplatelet | 22.01.2020 | 602 |
| 86 | m | 19.06.2020 | RCA prox | no | dual antiplatelet | 23.06.2020 | 4 |
| 72 | f | 01.03.2019 | RCA mid | no | dual antiplatelet | 19.04.2023 | 1510 |
| 58 | m | 03.05.2018 | LCA | no | single antiplatelet | 07.05.2018 | 4 |
| 89 | m | 12.04.2019 | LAD prox | yes | OAC + dual antiplatelet | 20.07.2022 | 1195 |

RCA: right coronary artery, LCX: left circumflex artery, LAD: left anterior descending artery

### Table S6 Univariable and Multivariable logistic regression results for risk factors of MACE in all AF patients (n=222)

|  | **Univariable Model** | |  | **Multivariable Model^a^** | |
| --- | --- | --- | --- | --- | --- |
| **Variables** | **OR (95% CI)** | **p-value^b^** |  | **Adjusted**  **OR (95% CI)** | **p-value^b^** |
| **Patient characteristics** | | | | | |
| **Age,** y (per unit increase) | 1.05 (1.02-1.09) | **<0.001** |  | 1.02 (0.98-1.06) | 0.246 |
| **Sex** (female) | 2.15 (1.14-4.04) | **0.018** |  | 2.10 (0.93-4.77) | 0.076 |
| **BMI** | 0.94 (0.86-1.03) | 0.199 |  | - | - |
| **Transthoracic echocardiography** | | | | | |
| **LVEF** | 0.99 (0.97-1.02) | 0.673 |  | - | - |
| **LA diameter** | 1.02 (0.96-1.07) | 0.549 |  | - | - |
| **Score** | | | | | |
| **GRACE Score** | 1.01 (1.00-1.02) | **0.007** |  | 1.01 (1.00-1.02) | 0.086 |
| **Vessel disease** | | | | | |
| **Single-vessel** | 0.71 (0.37- 1.36) | 0.309 |  | - | - |
| **Dual-vessel** | 0.62 (0.32-1.20) | 0.155 |  | - |  |
| **Triple-vessel** | 2.00 (1.11-3.60) | **0.021** |  | 2.34 (1.13-4.86) | **0.022** |
| ^a^ Multivariable model includes variables with p<0.05 on univariable logistic regression  ^b^ Pearson's Chi-squared test  CI: confidence interval; LA: left atrial; LVEF: left ventricular ejection fraction [%]; OR: odds ratio | | | | | |
